# How can humanitarian services provision during mass displacement better support health systems? An exploratory qualitative study of humanitarian service provider perspectives in Cox's Bazar, Bangladesh

**DOI:** 10.1016/j.jmh.2022.100132

**Published:** 2022-09-13

**Authors:** Sneha Krishnan, Samia Zaman, Muhammad Ferdaus, Md Humayun Kabir, Hafiza Khatun, SM Safiqur Rahman, Manar Marzouk, Anna Durrance-Bagale, Natasha Howard

**Affiliations:** a Jindal School of Environment and Sustainability, Jindal Global University, Haryana and Environment, Technology and Community Health Consultancy Services, Mumbai, Maharashtra, India; b BRAC University, UB04–66 Bir Uttam AK Khandakar Road, Dhaka 1212, Bangladesh; c University of Dhaka, Department of Geography & Environment, Nilkhet Road, Dhaka 1000, Bangladesh; d Saw Swee Hock School of Public Health, National University of Singapore and National University Health System, 12 Science Drive 2, Singapore 117549, Singapore; e London School of Hygiene & Tropical Medicine, Department of Global Health & Development, 15-17 Tavistock Place, London WC1H 9SH, United Kingdom

**Keywords:** Mass displacement, Refugees, Humanitarian response, Health systems strengthening

## Abstract

Health services provision in mass displacement settings is a humanitarian imperative and essential to promoting international and regional security. Internationally displaced populations experience a range of issues pre-, peri-, post-displacement and residing in host countries that affect their health and well-being. This study examined links between humanitarian and government health services provision for forcibly displaced Myanmar nationals (FDMN) in Cox's Bazar to consider how improved knowledge sharing and collaboration might better support health systems during mass displacement. We conducted a qualitative descriptive study, interviewing 25 humanitarian service providers in-person in Bangladesh in early 2021 and analysing data thematically. We found that government restricted what essential services humanitarian health actors could provide and FDMN had to undergo stringent screening and referral to receive tertiary healthcare. Concurrently, the government health system was challenged by accessibility, affordability and availability of medicines, equipment, and trained staff. Humanitarian health service providers augmented government responses by working with community groups, recruiting and training Rohingya volunteers, and involving religious leaders. Findings suggest that easing barriers to a fuller range of health services, allowing access to digital devices, and hiring FDMN to support their communities would improve health system responsiveness to the legitimate needs of FDMN displaced around Cox's Bazar. It is imperative to amplify and listen to the voices of FDMN and collaborate in addressing structural and social barriers constraining their access to effective health services, both to increase trust in and responsiveness of the health system.

## Highlights

• We examined links between humanitarian and government health services provision in Cox's Bazar, Bangladesh, interviewing 25 humanitarian service providers.
• The government health system is challenged by accessibility, affordability and availability of medicines, equipment, and trained staff.
• Humanitarian health service providers augmented government responses by working with community groups, recruiting and training Rohingya volunteers, and involving religious leaders.
• We urge all service provision organisations to recruit staff from FDMN communities and ensure Rohingya communities can engage in decision-making on health services provision.

## 1 Introduction

Health service provision for populations experiencing mass displacement is a humanitarian imperative and promotes national and regional security (Grove and Zwi, 2006). Internationally displaced people residing in host countries experience a range of issues pre-, peri- and post-displacement that affect their health and well-being (Greene et al., 2019). In host countries, especially in resource-poor regions, humanitarian health services provision can severely strain existing health infrastructure, impacting accessibility and affordability for both host and displaced populations (Newbrander et al., 2011). The most effective measures to prevent mortality and morbidity in such complex emergencies include protection from violence; provision of adequate food, clean water and sanitation; diarrhoeal disease control; measles immunisation; maternal and child healthcare, and case management of endemic infectious diseases (Toole and Waldman, 1997). Durrance-Bagale and colleagues have highlighted the need for co-ordinated, integrated, and co-operative responses at all levels (international, national, subnational) and by all actors (e.g. humanitarian system agencies, governments, civil society), contextualised to displaced and host population needs and socio-cultural realities (Durrance-Bagale et al., 2020). During the Covid-19 pandemic, along with direct health risks, indirect risks including disruption of camp supply chains, restructuring of humanitarian staffing and redirecting of resources to enable an adequate response could potentially overwhelm health systems and infrastructure in mass displacement settings (Lau et al., 2020).

The Rohingya people from Myanmar's Rakhine State have lived in refugee camps in Cox's Bazar, a coastal district of Bangladesh, since the 2017 forced exodus and an estimated 1.2 million – over a third of the population of Rakhine state – are now there (Mahmood et al., 2017). They have settled in collective sites (84%), collective sites within host communities (12%), and dispersed sites within host communities (4%) (IOM, 2019). The Bangladesh Ministry of Health and Family Welfare oversees the provision of medical services, providing dispensaries and clinics with staff who can perform minor surgeries within the camps (Rahman et al., 2020). Selected drugs are available without prescription from nearby shops, which are accessible to both host and Rohingya communities. Patients who require secondary or tertiary care are sent to local government medical college hospitals in Chittagong or Cox's Bazar (Rahman et al., 2020).

This qualitative study clarified links between humanitarian and government health services provision for forcibly displaced Myanmar nationals (FDMN) in Cox's Bazar by examining experiences of humanitarian actors providing health services in this setting. Objectives were to: (i) examine how health interventions by humanitarian actors influenced government health services provision; (ii) identify any examples of humanitarian actors collaborating with or enhancing health system capacities; and (iii) consider how improved knowledge sharing and collaboration might better support health systems during mass displacement.

## 2 Material and methods

### 2.1 Study design

We chose a qualitative design, involving semi-structured key informant interviews with national and international agency staff providing health and non-health services, to examine health system provisions for FDMN in Cox's Bazar, Bangladesh (Yin, 2018).

### 2.2 Research question

Our research question was: ‘How does humanitarian services provision during mass displacement affect health systems in Cox's Bazar?’

### 2.3 Sampling, recruitment and consent

SZ, MF, MHK and SMSR recruited interviewees purposively to cover a range of humanitarian clusters and agencies active in Cox's Bazar, namely International Organization for Migration (IOM) to discuss Camp Coordination and Management, Protection, and Shelter; World Health Organization (WHO) and Bangladesh Red Cross Society (BRCS) to discuss Health; United Nations Development Program (UNDP) to discuss Early Recovery/Disaster Management; United Nations Children's Fund (UNICEF) to discuss Education, Water, Sanitation and Hygiene, and Nutrition; World Food Program (WFP) to discuss Food Security and Logistics; and UN Women to discuss gender.

Interviewees were provided with a study information sheet and opportunity to discuss the study, and signed a consent form in Bangla or English before participation. Interviewees were anonymised via assigned code and classified by organisation and cluster for analysis (Table 1).

**Table 1:** Provider characteristics.

Code	Gender	Organisation	Cluster
EY	M	Danish Refugee Council	Camp Management
GX	M	IOM	Camp Management
HY	F	Danish Refugee Council	Camp Management
MY	F	IOM	Camp Management
FY	M	IFRC	Disaster Risk Management
BZ	M	Mukti	Education
GY	M	Mukti	Education
KY	M	UNICEF	Education
AX	M	UNFPA	Food
LY	F	WFP	Food
EX	F	UN Women	Gender
CX	M	UNICEF	Health
AY	F	Bangladesh Red Crescent Society	Health
BY	M	Bangladesh Red Crescent Society	Health
CY	M	Bangladesh Red Crescent Society	Health
DY	M	Bangladesh Red Crescent Society	Health
CZ	M	Action Against Hunger	Logistics
JY	M	UNICEF	Nutrition
BX	F	IOM	Protection
DX	F	UNICEF	Protection
DZ	F	BRAC	Protection
EZ	F	Relief International	Protection
FX	F	UNICEF	Protection
IY	M	IOM	Shelter
AZ	M	IOM	Shelter

NB: BRAC is Bangladesh Rural Advancement Committee; IFRC is International Federation of the Red Cross; IOM is International Organization for Migration; UNFPA is United Nations Population Fund; UNICEF is United Nations Children's Fund; WFP is World Food Programme.

### 2.4 Data collection

We developed the interview guide iteratively to include: communication with and participation of vulnerable FDMN in service provision, co-ordination measures, barriers and enablers to providing services to FDMN, and links between humanitarian health services and government health support provided for FDMN living in Cox's Bazar.

SZ, MF, MHK and SMSR conducted semi-structured interviews with humanitarian service providers working in Cox's Bazar between December 2020 and February 2021 in Bangla or English. We judged that saturation had been reached when no new information or opinions were shared by interviewees. All interviews were audio recorded and ranged 40-65 min in length. SZ transcribed all audio recordings and translated interviews from Bangla into English as necessary.

### 2.5 Analysis

SK used a reflexive thematic analysis approach (Braun and Clarke, 2006) to code and identify themes in interview transcripts using NVivo 12 data analysis software (QSR International Pty Ltd. Version 12, 2018). Inductive themes were presented under four overarching topics: (i) health services provision for FDMN; (ii) coordination and linkages between humanitarian and government health responses; (iii) barriers to health services provision for FDMN; and (iv) enablers to health services provision. All authors discussed and agreed themes iteratively before SK coded transcripts in full.

### 2.6 Ethics

Research ethics committees at the University of Dhaka (reference 3/58863-65) and London School of Hygiene and Tropical Medicine (reference 17274) provided approval.

## 3 Results

### 3.1 Interviewee characteristics

Table 1 presents characteristics of the 25 interviewees, 15 men and 10 women.

### 3.2 Health services provision for FDMN

Sub-topics included: (i) available health infrastructure and services for FDMN; (ii) gaps in health services provision; and (iii) shared Covid-19 responses.

#### 3.2.1 Available health infrastructure and services for FDMN

Basic health infrastructure in the refugee camps consisted of a health post and hospital run by BRCS, with inpatient, outpatient and outreach services for FDMN. Interviewees considered outpatient health services accessible for FDMN, as they were situated within camps. Some reported that their organisations provided both inpatient and outpatient services, but most focused on outpatient services and referred complicated cases to the district hospital.

*‘The nearest camp settlement is within 5 to 6 feet from our facility. So, it's very near to the camp. We provide inpatient and outreach service. Inpatient service is not available in our hospital.’* [AY, BRCS]

FDMN underwent a screening and referral process to access tertiary health services at the government health centre.

‘*NGOs [non-governmental organisations] referred severe health-related issues to government hospitals at the upazila* [sub-district] *level, district or divisional level. But to visit these hospitals FDMNs need to take permission from the Camp in-Charge which is a very long and rigid process.’* [AY, BRCS]

Interviewees commented on different types of services required by different FDMN groups (e.g. women, children, the elderly) and described their overall health requirements. Common conditions included diabetes, hypertension, skin diseases, peptic ulcers, mental trauma, and sexually transmitted diseases. Some raised concerns about the spread of infectious diseases caused by poor living conditions and overcrowded camps.

*‘As they are living in congested areas contagious diseases spread easily and quickly[...]. Government should take steps to improve the environment and infrastructures of the camp area as some diseases are occurring due to living in congested areas and unhealthy environments.’* [BY, BRCS]

Some organisations provided door-to-door counselling and hygiene kits to women, who were uncomfortable about requesting essential items from the health centre due to the stigma attached to discussion of personal hygiene needs.

*‘Most of the women suffer from vaginal discharge due to an unhygienic lifestyle. We counsel them and provide them hygiene kit. It consists of a towel, soap, and a container.’* [BY, BRCS]

Immunisation, family counselling and children's physical and mental health requirements were identified, assessed, and monitored through case management by camp volunteers, who provided health services accordingly.

#### 3.2.2 Gaps in health services provision

Despite the apparent accessibility of health facilities, there were concerns about lack of amenities and basic equipment in community health posts. Service providers mentioned shortages of medical equipment and medicines in the camps. A frequent example was patients not being able to get prescribed medicines.

*‘We have a shortage of medicines, that's why after diagnosis of the disease we cannot supply the prescribed medicines to patients. NGOs only stock and supply some common medicines which actually don't provide much benefit to the patient.’* [CY, BRCS]

*‘There is no well-equipped diagnostic centre, laboratory and specialised centre for surgery. So, sometimes it becomes difficult to diagnose diseases like diabetes.’* [AY, BRCS]

Despite registering complaints and approaching higher authorities for action, health service providers noted the lack of accountability and financial resources to support delivery of medicines for FDMN. This reportedly reflected a typical top-down approach in humanitarian management, which excluded inputs from frontline staff and communities.

*‘We submit our complaint to the management regarding these problems, but the management hasn't yet taken any steps to solve these issues. Head of operation, Deputy Director and other authoritative persons don't organize any meeting with us and don't take any feedback and opinion from us. They pay visits to the hospital but consult only with the management.’* [CY, BRCS]

Several organisations provided counselling to FDMN within the camps, mostly with the help of community nurses who were directly consulted in case of medical issues. Providers reported encouraging family planning among FDMN but suggested this was largely ignored due to ‘conservative attitudes’.

*‘But women don't want to take contraceptives and men don't want to use condoms. The FDMN community is very conservative in case of family planning. Steps should be taken to solve this problem [...]. The main concern should be family planning as the birth rate of the Rohingya community is increasing day by day, data showed that in 2017-2018 almost 35 to 36 thousand deliveries took place.’* [BY, BRCS]

Adequately trained staff and specialist care providers were lacking in the camps. While some NGOs brought their own medical staff, there were not enough to provide for the numbers of people in the camps.

#### 3.2.3 Shared Covid-19 responses

Humanitarian health providers initiated awareness interventions in 2020 to prevent the spread of Covid-19 into the camps. An IOM interviewee noted the use of community radio to disseminate Covid-19 prevention strategies, while awareness targeted social groups such as religious leaders and mothers’ groups to enhance communication within host and FDMN communities.

Views of the government response to Covid-19 were mixed. A few interviewees praised the health ministry's provision of guidelines and awareness raising. Others described hospitals creating isolation units for suspected Covid-19 patients. Any FDMN testing positive for Covid-19 was referred to hospital immediately. However, one noted that FDMN were less likely to be infected with Covid-19 than people living in the host community [BY, BRCS]. Other interviewees described challenges.

*‘The health sector gave us so many guidelines to follow to ensure camp health safety, and those guidelines are ensured to be followed strictly. The health-worker and field-worker are working hard here. We should focus on ensuring the safety of them. Apart from this, we have no gaps here.’* [BZ, Mukti]

*‘I think the health system for FDMNs is still not much developed. For example, during this pandemic we couldn't provide enough health services to the FDMNs and panic regarding Covid-19 is the main reason for it.’* [HY, Danish Refugee Council [DRC]]

Interviewees noted that promoting adherence to safe distancing norms was challenging in overcrowded, densely populated camps. When they visited health centres there was no social distancing.

*‘When the FDMNs get information that a specialist doctor will visit any field hospital, 3 to 5 thousand people gather around the hospital, and they don't maintain any kind of social distance.’* [LY, UNWFP]

### 3.3 Coordination and linkages between humanitarian and government health responses

An interviewee clarified the role of the department of health in managing health services provision in Cox's Bazar.

*‘Government is providing an oversight at the policy level and on the technical documents through the management information system which is aligned between the camps under the host community. The government is also having a role of the quality controller for the medical supplies which are being brought by the different organizations. Under the provision of universal health the government has a role of overseeing, quality controlling, leadership, internal policy adviser etc. So, all these NGOs which are working inside the camp are also directly or indirectly engaged and involved with the office of the civil surgeon and provision of the health services at the host community through the government and this coordination mechanism is bringing together the linkages.’* [JY, UNICEF]

Humanitarian interviewees discussed their perceptions of working with the government health system, with one describing coordination between humanitarian and government health service provision.

*‘In the host community, health services are provided by the government and in the camp settlement most of the health services are provided by the NGOs. INGOs and the UN agencies and the government overview the services. So, the linkage I see is the coordination mechanism, especially the health sector coordination.’* [JY, UNICEF]

Some organisations tried to augment government health services by coordinating with relevant ministries, and building government staff and community capacities.

*‘We are engaging in all these activities while working with the government. In case of health service, the government is much stronger than the health services that the NGOs are providing in the camp in terms of their capacity, provision services, roles that they are playing in the host community. UNICEF is involved with the Ministry of Education, Ministry of Water and Sanitation, we are working with all of these organizations. We are also working at the local level of Cox's Bazar for capacity-building activities or we are helping the government to organize it.’* [JY, UNICEF]

Links with the government health system were seen as crucial for sustaining operations. Some interviewees suggested that government restrictions on provision of services for FDMN should be resolved with dialogue and communication.

*‘In our project proposal we plan to provide various services to the FDMNs. But sometimes we cannot implement those plans as we don't get permission from the government and CiC [Camp in-charge]. Government controls all the camps.’* [HY, DRC]

An interviewee working in the Protection cluster described efforts to create an enabling environment through integration of humanitarian and government health systems.

*‘We are working for child protection, sometimes we get some children who are seriously ill or injured and they need to be referred to the health services and we follow up the condition to know whether it is working well or not. Though we refer them to health centres sometimes they don't get the proper treatment. In that case, the health centres refer them to the government hospitals. For major surgeries the FDMNs are referred to the government hospitals like Cox's Bazar medical hospital, Chittagong medical hospital.’* [EZ, Relief International]

Another noted the limitations of working within government-mandated health policies.

*‘Government controls all the camps. So, we cannot provide any service without the permission of the government. Sometimes even if we want to provide some services which we think are essential we cannot provide those services if the government thinks that providing those services will be harmful for our country. So, we need more independence and flexibility.’* [EY, DRC]

Coordination between government and non-governmental health services appeared especially effective for vaccination.

*‘When we started our vaccination program, we contacted the chief of every organisation and tried to make them understand the priority and desire of the government. At the beginning we faced some difficulties but gradually all the organisations cordially accepted us and participated in our weekly coordination meetings, and they also sent participants like maternity service providers, midwives in different training and orientations which are arranged by us. They even attend training that we organize in Sadar Hospitals. So, the linkages between health services delivered by the government and health and other services provided for FDMNs work well.’* [CX, UNICEF]

Two interviewees suggested that better government health staff attitudes toward FDMN would earn their trust and improve their experience of accessing health centres. Improving existing health service provision for FDMN involved increasing supply of necessary drugs, providing skilled and qualified personnel, and enhancing community capacities.

*‘Health sector service providers should be more friendly and supportive towards the community people. The FDMNs have to wait in a long queue to get treatment and the place becomes chaotic. There is no systematic way of maintaining the queue. Only a minimum package of health service is provided to the community people. So, the health services are not sufficient for demand. The doctors are not enough qualified and the organizations sometimes recruit nurses and midwives from the host community who don't even have any specialised degree.’* [EY, DRC]

*‘Some FDMNs visit several field hospitals for the same disease even after getting treatment. Sometimes the doctors feel irritating as they have to work for long hours. The standard of the government health system and the health system of the FDMNs is not the same. Some organizations provide good health services but some organizations don't. Also, the behaviour of all the staffs is not the same. There is also lack of cordiality and sincerity.* [IY, IOM]

As camps were governed and managed by the Bangladesh military and paramilitary forces, one interviewee suggested political changes would improve the lives of FDMN.

*“We are trying to coordinate with different sectors and with the government. The camps should be governed by the elected bodies instead of army persons and we should also ensure the participation of the women in leadership positions”* [EX, UN Women]

Others suggested changes could be achieved through advocacy and dialogue between government authorities and FDMN communities.

*‘Humanitarian space has shrunk over time [...]. the camps are congested, there are limitations, and given the resources that are available from the humanitarian community there are also practical limitations about what we can do to assist them [FDMN]. Our best way of trying to address the barrier is through advocacy or continuous dialogue with authorities and other influential people in the community, so that the humanitarian space is not further eroded and no further harms come to the Rohingya refugees.’* [BX, IOM]

### 3.4 Barriers to providing services for FDMN

Interviewees described multiple barriers. The main barrier highlighted was the physical risk inherent in camp locations. FDMN were exposed to fires, floods, cyclones and accident risks due to their precarious location in hilly, congested areas. Overcrowding and resource limitations worsened existing risks and acted as additional barriers to providing and accessing health services. For example, people with disabilities found it difficult on the uneven and often flooded terrain and organisations were unable to provide accessible facilities due to these logistical challenges.

Language remained a major barrier, as most communications were disseminated in Bangla or English. Interviewees suggested this could be resolved by recruiting Rohingya or people from host communities, as the local Chittagonian dialect was very similar to Rohingya dialect. Several organisations appointed translators, which helped somewhat.

Gender norms related to women and girls among FDMN presented barriers to education, especially when trying to ensure inclusion. Interviewees suggested that women in FDMN communities were vulnerable and less vocal than men during meetings, due to limited education, awareness, and encouragement. At the same time, some organisations recruited women volunteer coordinators to work with women, children, and the elderly as they were likely to be better accepted among these subpopulations.

Early marriage, especially for girls, was reportedly frequent in the FDMN community and restricted girls’ access to education. Interviewees described parental attitudes toward girls’ education as shifting once girls reached 10 years old, as this was the age to leave education and get married.

*‘Rohingya people tend to lock their female child if they reach the 10-year-old age, like saying, these are enough for you, and you have no need to take more education. It's a common scenario at the camp.’* [BZ, Mukti]

FDMN digital literacy and access to devices such as mobile phones was limited, compounded by government restrictions on using local SIM cards and poor internet availability within camps. Some interviewees suggested that if the government provided internet connectivity for FDMN communities, organisations could provide internet-based learning and other support for young people.

Barriers were noted among different faith communities sharing the camps, including Muslim, Buddhist, Hindu and Christian. Interviewees described effects of longstanding traumas and trust issues between communities.

*‘The FDMNs are very much conservative, especially in the case of religion [...]. For example, if we arrange any event and provide food, the Hindu community doesn't take the food from us as we are from different religions [...]. The Muslim community doesn't like Buddhist community as they have been abused by the people of this religion. The Muslim community also has some trust issues as they have gone through trauma and since they are living in our country.’* [DZ, BRAC]

Insufficient funds posed another significant barrier to health services provision. International donor funding to support FDMN was insufficient, which directly affected services.

*‘We used to provide BDT 1000 per person to the FDMNs, but now due to shortage of funds we are providing them only BDT 931 and this is not sufficient to purchase rice, pulse, oil and other groceries. Moreover, the price of groceries is increasing day by day. Our organisation is trying to ensure everyone is taking at least 2200 kilocalories, but I don't think we are fully successful in this regard.’* [LY, UNWFP]

Interviewees reported that domestic and international NGOs generally only provided food and other non-financial support to FDMN. Consequently, many FDMN sold these items when they needed money.

*‘So, they sell out the reliefs in the local markets…’* [CX, UNICEF]

Some reported thefts in terms of ingratitude rather than desperation or essential pragmatism.

*‘We provided solar panels and solar lights in the toilets of the FDMNs and a few days later they stole away all the solar panel and solar lights, so they still don't know what is good and what is bad for them.’* [CZ, Action Against Hunger]

Others suggested such issues could be resolved by including FDMN communities in programme design, to ensure they could get what they actually needed, or allowing FDMN to work and create a ‘source of income’.

### 3.5 Enablers to providing health services for FDMN

Interviewees described several cross-sectoral enabling factors that facilitated health service provision and access in the camps. Interviewees mentioned cooperation and coordination between camp volunteers, organisations, government, host community and FDMN groups. For example, several non-health sector organisations provided emergency ambulances and maternal and child services.

Several described how working alongside government helped build capacity and push forward the localisation agenda.

*‘The policies and the strategies of the government and the UN agencies on the localisation of the strategies or building the capacity of the local NGOs and delegating the responsibility to the ground level and the monitoring is happening through the CiC and the UN agencies.’* [JY, UNICEF]

A key enabler was engaging with traditional leaders, such as imams and muezzins (male religious leaders) in Rohingya communities. As they were respected within communities, they were instrumental in spreading health promotion messages and could act as a link between FDMN, service providers, and the government.

*‘The religious groups helped us in passing the messages including the message of Covid-19 as the Rohingya people listen to religious leaders more than the others. We are working with all these imams for community engagement and community education and helping us in bringing together the adolescents and the youths in our programmes.’* [JY, UNICEF]

However, some interviewees noted that issues of power and marginalisation within communities needed to be considered when working with any community elites, such as religious leaders. For example, religious leaders were not noticeably active against gender-based violence or in protection.

*‘Women are very much vulnerable in the camp. Rape, sexual violence, polygamy is increasing day by day in the camps and they [religious leaders] sometimes don't speak about it*.’ [DX, UNICEF]

One interviewee cautioned that religious leaders could take advantage of their authority.

*‘There are few groups or few people those are using their power like imam. They are the big boss in the camp, so sometimes it's difficult to communicate with the general refugees because sometimes their voices are suppressed under the power [...]. They get biased like we want to distribute anything they try to favour those people who are close to them.’* [EX, UN Women]

Several interviewees suggested that improved communication and community engagement had enabled them to provide better services. Committees for the elderly, women and young people adopted inclusive approaches, including use of a community feedback and response mechanism, again reflecting localisation agenda efforts within the humanitarian sector to put FDMN at the centre of service delivery. Community engagement was thus an essential enabler to allow service providers to understand and respond to community needs.

*‘We always consult with the community before providing any service to find out their actual needs. We also provide them instruction on how to use the services that we are providing. We also take feedback from them, which helps us to identify their wants, what they need, how much they need etc. It gives us support to make best use of our limited resources and reduces wastage. I think this is the main supporting factor.’* [MY, IOM]

## 4 Discussion

This study was a first effort to understand links between humanitarian and government health service provision, which is crucial for sustaining operations in protracted mass displacement settings such as the FDMN camps around Cox's Bazar. We describe how health interventions by humanitarian actors could potentially better support government health services provision. For example, although government and humanitarian health service provision catered to both FDMN and host populations around Cox's Bazar, government services were only available at sub-district level. The additional financial, logistical, and physical burdens on FDMN often prevented them accessing critical health services, as similarly described for Syrian refugees unable to afford high-priced care for non-communicable diseases in Jordan (McNatt et al., 2019). Some interviewees suggested that government restrictions on provision of services for FDMN should be resolved with dialogue, improving supplies, recruiting qualified personnel, and enhancing community capacities. However, given ongoing funding restrictions for FDMN support, it remains unclear how this will be enacted in practice. Coordination between government and non-government health services provision appears to have been most effective for vaccination programmes, in Cox's Bazar and elsewhere (Jalloh et al., 2019, 2020).

Due to the mass cross-border Rohingya displacement, the health system was challenged by a double service provision burden for both Bangladeshi population and arriving FDMN. This was worsened as both populations were threatened by the COVID-19 pandemic. With limited availability of resources in camps, health-worker capacities were stretched just trying to provide essential services, as seen in contexts such as South Sudan (Jones et al., 2015). Strengthening health services provision thus requires active engagement with communities in planning and decision-making. Religious leaders are able to help, particularly in health promotion and outreach (e.g. increasing awareness of Covid-19 prevention and vaccination) but socially marginalised community groups must also be involved (Jalloh et al., 2019, Lindsjö et al., 2021).

The impact of Covid-19 on health programming was predictable and similar to that in other displacement settings such as Syria (Marzouk et al., 2022). Interviewees noted limited adherence to Covid-19 preventive measures, and limited capacity to test or isolate potential Covid-19 cases in the camps (Rahaman et al., 2020). Shared sanitation facilities and overcrowded shelter infrastructure in displacement camps make safe distancing extremely difficult, as also found in Syria (Barua and Karia, 2020; Krishnan, 2020; Douedari et al., 2020). Other impacts included increased incidence of child marriage, as found in other settings (Nagashima-Hayashi et al., 2022), and potentially exacerbated by reduced availability of reproductive and sexual health counselling services (Guglielmi et al., 2020; Melnikas et al., 2020).

The dynamic needs of FDMN, particularly more vulnerable sub-groups, require monitoring for emerging health risks. Health impacts of mass displacement were worsened by exposure to natural hazards, overcrowding, poor nutrition and shelter, and marginalisation within camp settings, particularly for pregnant women, children, and disabled and elderly people (Zaman et al., 2020). However, resolving these issues requires structural and policy change, particularly given donor fatigue and constrained funding. Research on early recovery from disasters shows that short-term initiatives are ineffective in addressing pre-displacement vulnerabilities (Sadik et al., 2018). Thus, for example, addressing unmet reproductive health needs for displaced women requires longer-term measures supporting gender equality, as was demonstrated in Colombia (Rivillas et al., 2018). Recent research conducted in Bangladesh with 400 FDMN women underlines the urgent need for sexual health education in this community. Knowledge around family planning was lacking and women believed that their husband had to give permission for them to use birth control, although 40% felt unable to discuss these issues with their husband (Azad et al., 2022). Another study in Kutupalong camp in Cox's Bazar found a positive association between women's use of contraceptives and the presence of a health centre in the camp. Women employed outside the home were also more likely to use contraceptives (Khan et al., 2021).

This study provides lessons from the humanitarian sector to help improve health system responses to mass cross-border displacement. These include the value of stronger links between humanitarian and government health services, going beyond coordination and referral to focus on making healthcare equally accessible, affordable, and equitable for displaced and host communities. There are practical benefits to engaging religious leaders, requiring further research to understand both opportunities and risks. Cash transfers were reportedly not used in Cox's Bazar, but a recent scoping review showed these have been successful in supporting livelihoods during displacement (Durrance-Bagale et al., 2020) as also shown in Yemen (Ecker et al., 2019). This lack of flexibility from NGOs is reflective of a top-down approach to humanitarian management, while the alternative of engaging displaced communities and working with religious leaders - as described by several interviewees - aligns better with the Grand Bargain, a commitment by donors to ensure those receiving aid have a voice in decisions that affect them (Inter-Agency Standing Committee 2020). Global health agendas and policies offer protection to only a small proportion of the world's population, while our connectedness has become more visible during the Covid-19 pandemic and new and ongoing conflicts globally, thus improving health services provision and support for forcibly displaced people as part of humanitarian planning and reform is a clear priority (Alawa et al., 2020).

Our policy recommendations are that recruitment of staff from FDMN communities should be a priority; local communities should be actively engaged in discussion of services to ensure services are appropriate and communities are not negatively affected; coordination, collaboration and knowledge-sharing between government and non-government health services should be encouraged and developed, potentially by regular meetings between actors involved in both types of service. These actions will help to improve effectiveness and build trust between all actors.

Although the study sample was designed to include government representatives, most were unable to participate due to competing Covid-19 priorities. This study thus reflects the perceptions and experiences of humanitarian service providers in Cox's Bazar and is not intended to be generalised to other contexts. Future research would benefit from more diverse perspective on knowledge and cross-organisational learning. However, we anticipate that our findings will have relevance for other mass cross-border displacement settings.

## 5 Conclusions

This study examined links between humanitarian and government health services provision in Cox's Bazar to consider how improved knowledge sharing and collaboration might better support health systems during mass displacement. Based on our findings, we urge the government of Bangladesh to reduce barriers to provision of needed health services, to allow recruitment of FDMN staff, and to enable FDMN access to digital devices as initial ways of improving living conditions in Cox's Bazar camps. We similarly urge humanitarian and government actors to engage with FDMN communities and listen to displaced people, both to better understand constraints to health services access and usage and to build mutual trust and respect between healthcare providers and displaced and host community service-users.

## Author contributions

NH, SK, MF and MHK conceived the study, with inputs from HK and ADB. MF, HK, SMSR and SZ collected data. SZ transcribed and translated interviews with inputs from MF and HK. SK analysed data and drafted the manuscript with support from SZ, MF, MM and NH. ADB and NH revised for critical content. All authors contributed to interpretation and approved the final version for submission.

## Funding

This study was supported by an MRC Health Systems Research Initiative Foundation study synthesising evidence from other sectors to strengthen health system responses to mass displacement (MR/S013008/1) awarded to NH. The funder had no involvement in research conduct or manuscript preparation.

## Declaration of Competing Interest

The authors declare that they have no known competing financial interests or personal relationships that could have appeared to influence the work reported in this paper
